# The secret life of memory receptors

**DOI:** 10.7554/eLife.71178

**Published:** 2021-07-14

**Authors:** Hovy Ho-Wai Wong, Olivier Camiré, P Jesper Sjöström

**Affiliations:** 1 Centre for Research in Neuroscience, Department of Medicine, The Research Institute of the McGill University Health CentreMontrealCanada

**Keywords:** CA3, ionotropic, hippocampus, presynaptic calcium, autoreceptors, Mouse, Rat

## Abstract

The canonical hippocampal NMDA memory receptor also controls the release of the transmitter glutamate and the growth factor BDNF.

**Related research article** Lituma PJ, Kwon HB, Alviña K, Luján R, Castillo PE. 2021. Presynaptic NMDA receptors facilitate short-term plasticity and BDNF release at hippocampal mossy fiber synapses. *eLife* **10**:e66612. doi: 10.7554/eLife.66612

The human brain contains around 86 billion neurons that communicate with each other through electrical and chemical signals. In the signaling neuron, an electrochemical event known as an action potential, or spike, triggers the release of molecular messengers into the synaptic cleft between two connected neurons. These neurotransmitters are then detected by postsynaptic receptors in the recipient cell. As information in the brain generally flows from the pre- to the postsynaptic neuron, it might seem unlikely to find any neurotransmitter receptors on the presynaptic, transmitting side (Figure 1A).

**Figure 1.**
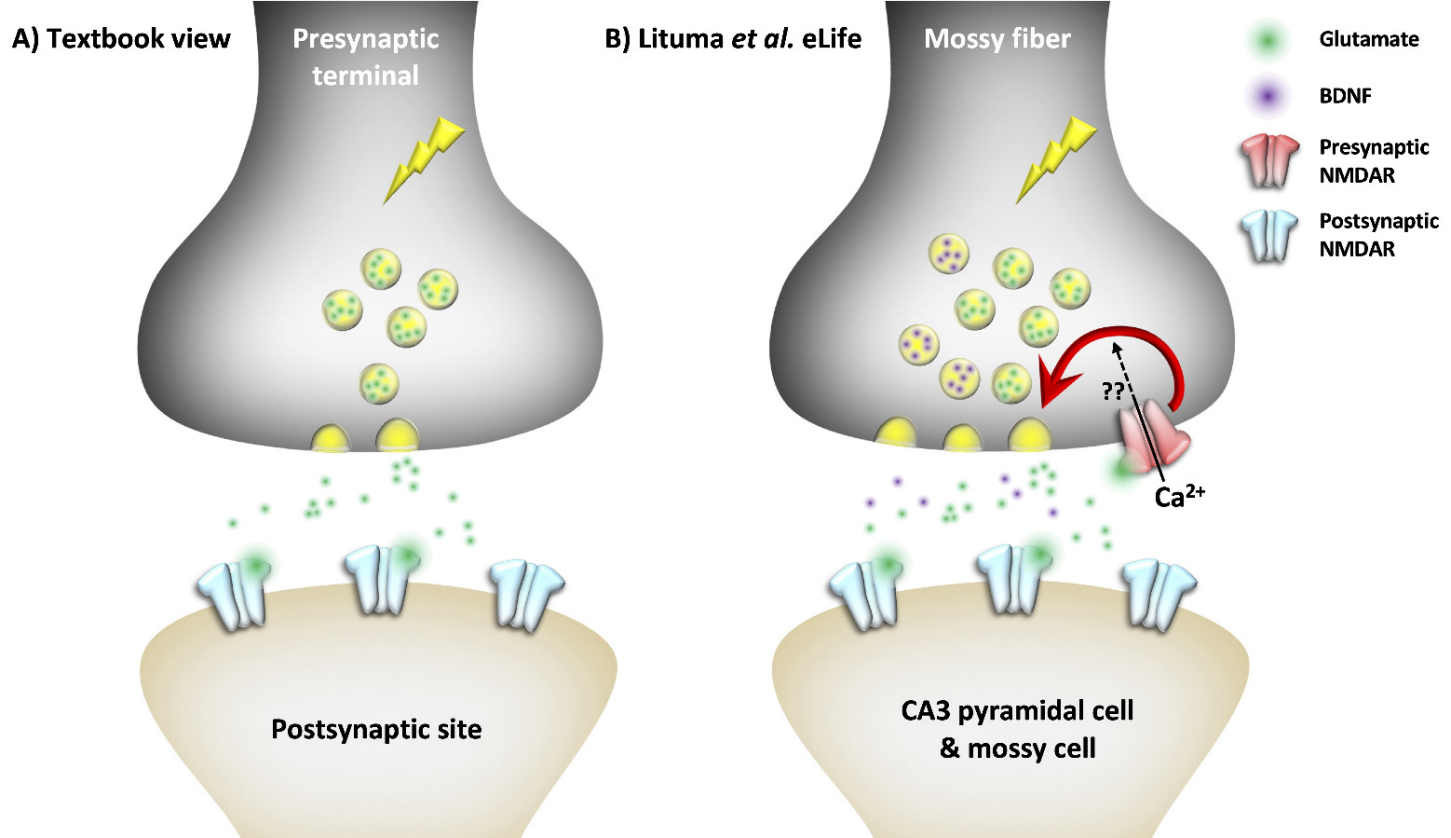
Presynaptic NMDA receptors regulate synapse-typespecific neurotransmission. (**A**) In the textbook view of central neurotransmission, the presynaptic spike (lightning symbol) elicits the release of a neurotransmitter (e.g., glutamate; green), which binds to postsynaptic glutamate receptors such as NMDARs (blue). (**B**) However, Lituma et al. found that presynaptic NMDARs (red) in hippocampal mossy fibers facilitate the release of glutamate (green) and a growth factor called BDNF (purple), possibly through an influx of calcium ions (Ca^2+^; question marks). The released glutamate may further activate presynaptic NMDARs (red) in a form of loop.

Yet, early electron microscopy studies revealed that N-methyl-D-aspartate receptors (NMDARs) – which are glutamate receptors and ion channels – are present on both pre- and postsynaptic neurons (e.g., Siegel et al., 1994). NMDARs on postsynaptic cells play an important role in memory formation and Hebbian plasticity — that is, the strengthening of the connections between presynaptic and postsynaptic neurons that are activated together. However, their roles on the presynaptic side remain hotly debated (Wong et al., 2021). Now, in eLife, Pablo Castillo and colleagues at the Albert Einstein College of Medicine and the Universidad Castilla-La Mancha – including Pablo Lituma as first author – report how presynaptically located NMDARs (preNMDARs) are involved in regulating the release of the neurotransmitter glutamate (Figure 1B, Lituma et al., 2021).

Lituma et al. used electron microscopy to examine whether NMDARs are located on the axons of granule cells in the rat hippocampus, known as mossy fibers. These axons help to encode contextual and spatial memory by forming the main information pathway from the dentate gyrus to the CA3 region of the hippocampus, where they contact both excitatory pyramidal neurons and inhibitory neurons (Rebola et al., 2017). The electron microscopy results revealed that 32% of NMDARs were indeed present at the presynaptic sites of neurons.

To identify the purpose of these preNMDARs, the researchers explored low-frequency facilitation, a form of short-term plasticity specific to mossy fiber synapses. As expected, stimulation at 1 Hz temporarily strengthened the mossy fiber connections onto CA3 neurons in mouse brain tissue. However, pharmacologically blocking the receptors, or selectively deleting them through genetic engineering, reduced low-frequency facilitation, indicating an involvement of preNMDARs. Further experiments confirmed that this phenomenon was mediated by preNMDARs present in axons of the transmitting neurons, rather than NMDARs located in their cell bodies or dendrites.

Next, Lituma et al. wanted to test whether preNMDARs could contribute to synaptic facilitation due to high-frequency activity patterns that are more physiologically relevant. Therefore they stimulated mossy fibers using optogenetics and electrophysiological methods to mimic the brief bursts of action potentials seen in granule cells of the intact brain. Indeed, connections between mossy fibers and CA3 neurons were strengthened during these brief bursts. In contrast, removing or blocking preNMDARs reduced this burst-induced facilitation as well as the ability to evoke postsynaptic spiking responses. Thus, preNMDARs are pivotal for boosting synaptic information transfer.

It is possible that preNMDARs could contribute to glutamate release by boosting presynaptic calcium signals. To test this hypothesis, Lituma et al. monitored calcium levels using an imaging technique called 2-photon microscopy. This showed that upon burst firing, only neurons with intact preNMDARs saw boosted calcium signals in their mossy fibers. Additional experiments confirmed that a glutamate-induced rise of calcium ions only took place if NMDARs were present on the mossy fibers. This shows how preNMDARs promote calcium influx into mossy fibers, which could in turn enhance short-term facilitation.

Lituma et al. further speculated that the influx of calcium may additionally trigger the release of brain-derived neurotrophic factor, or BDNF — a growth factor involved in long-term plasticity and memory (Alonso et al., 2002; Kang and Schuman, 1995). Although a direct participation of preNMDAR-mediated calcium signaling remains to be confirmed, preNMDARs were found to be important for BDNF release.

In summary, Lituma et al. have provided compelling evidence that the preNMDARs present in mossy fibers contribute to synaptic information transfer. Interestingly, they also found that this role of preNMDARs was restricted to a subset of mossy fiber synapses, which was determined by the target neuron type: preNMDARs facilitated inputs to CA3 pyramidal neurons and to mossy cells, but not those to inhibitory neurons.

Still, some mysteries remain. For example, NMDARs have a well-known dual need for presynaptically released glutamate and postsynaptic depolarization to activate and elicit the calcium signals that in turn trigger long-term plasticity. This feature makes postsynaptic NMDARs ideal as coincidence detectors in Hebbian learning, which is triggered by simultaneous activity in connected cells. But when situated presynaptically, this dual need seems to make preNMDARs hard to activate — the spike that causes the glutamate release only lasts a millisecond, so the depolarization is long gone by the time preNMDARs become glutamate bound. So how are preNMDARs activated?

One possible answer is high-frequency presynaptic firing, during which subsequent spikes in a burst depolarize glutamate-bound preNMDARs (Abrahamsson et al., 2017). This, however, seems unlikely to happen during low-frequency facilitation at 1 Hz. Alternatively, these preNMDARs may also signal by changing conformation when binding glutamate – without the need for depolarization or calcium flux – similar to postsynaptic NMDARs in the hippocampus and preNMDARs in neocortex (Abrahamsson et al., 2017; Dore et al., 2016).

Intriguingly, flux-independent NMDAR signaling has been linked to Alzheimer’s disease while BDNF has been linked to epilepsy, which could make preNMDARs potential therapeutic targets (McNamara and Scharfman, 2012; Dore et al., 2021). Moreover, the synapse-type-specific regulation could potentially be leveraged for drug specificity. While many questions surrounding preNMDARs are yet to be answered, Lituma et al. provide exciting new evidence to unveil the secret life of NMDARs.
